# Supplementary material for: Prostate-Specific Antigen Monitoring Using Nano Zinc(II) Metal–Organic Framework-Based Optical Biosensor
Source: Biosensors (Basel). 2022 Oct 27;12(11):931. doi: 10.3390/bios12110931 (PMC9688191; doi:10.3390/bios12110931)
Supplement: Supplementary file 1 [file biosensors-12-00931-s001.zip › biosensors-1981052-supplementary.pdf]

Supporting Information

for

Prostate-Specific Antigen Monitoring Using Nano Zinc(II) Metal–Organic Framework-Based Optical Biosensor

Said M. El-Sheikh [1,*], Sheta M. Sheta [2,*], Salem R. Salem [3], Mohkles M. Abd-Elzaher [2], Amal S. Basaleh [4] and Ammar A. Labib [2]

1 Department of Nanomaterials and Nanotechnology, Central Metallurgical R & D Institute, Cairo 11421, Egypt
2 Department of Inorganic Chemistry, National Research Centre, Cairo 12622, Egypt
3 Department of Biochemistry, Egypt Centre for Research and Regenerative Medicine, Cairo 11887, Egypt
4 Department of Chemistry, Faculty of Science, King Abdulaziz University, P.O. Box 80203, Jeddah 21589, Saudi Arabia
* Correspondence: selsheikh2001@gmail.com (S.M.E.-S.); dr.sheta.nrc@gmail.com (S.M.S.); Tel.: +20-1022316076 (S.M.E.-S.); +20-1009697356 (S.M.S.); Fax: +20-227142451 (S.M.E.-S.); +20-233370931 (S.M.S.)

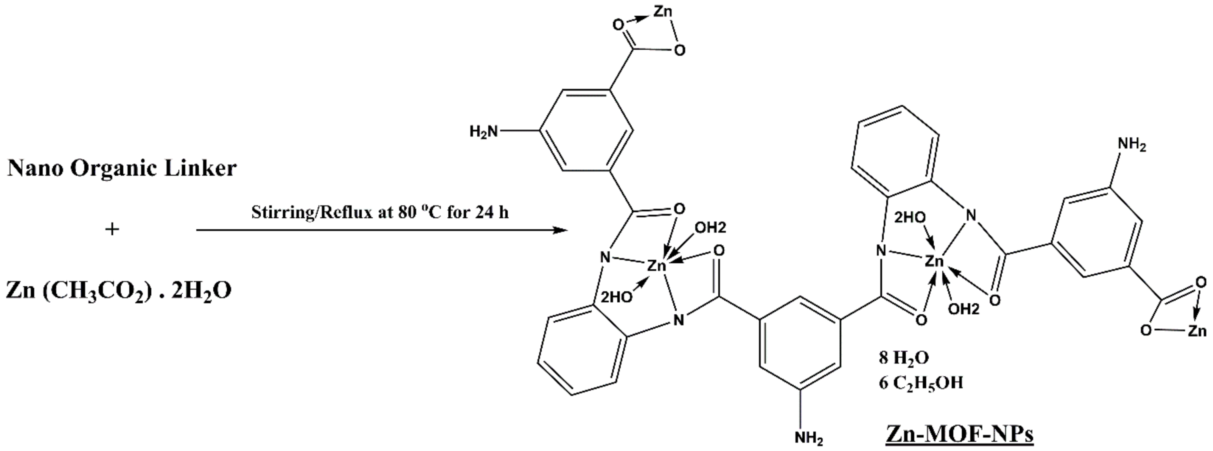

Fig. S1: The proposed mechanism of the Zn-MOFs-NP synthesis.

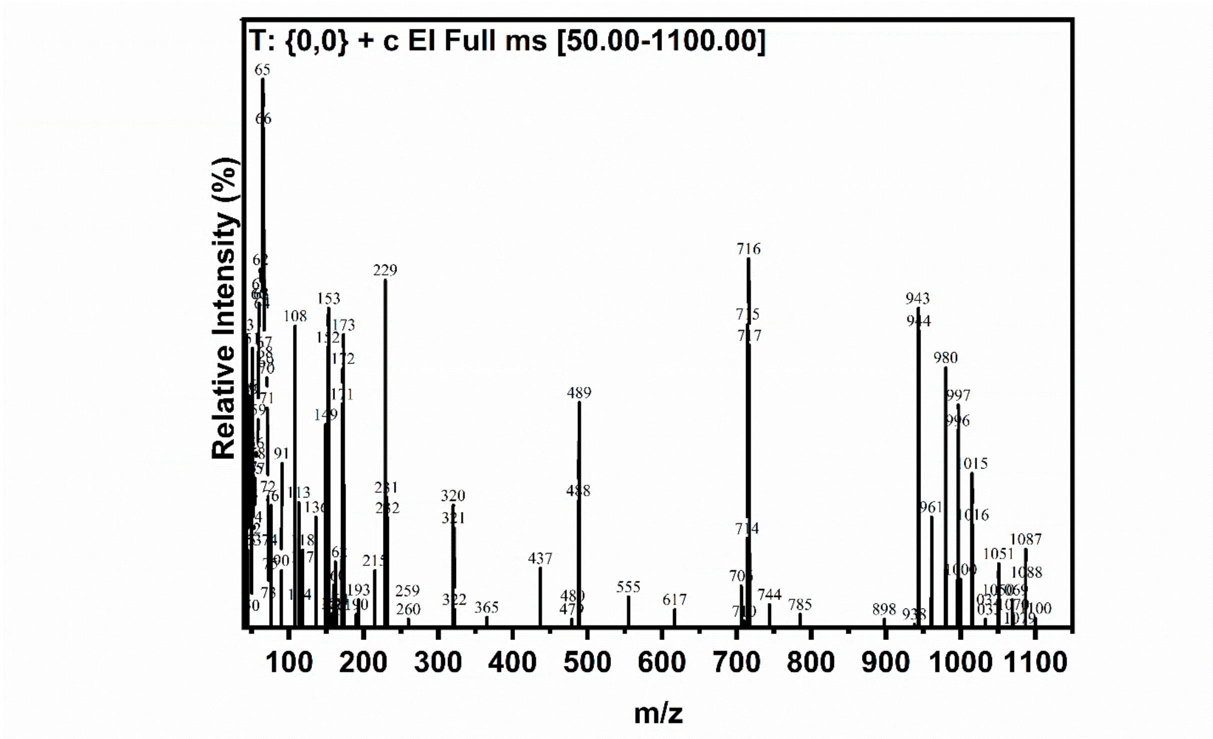

Fig. S2: Mass spectrum of the Zn-MOFs-NP .

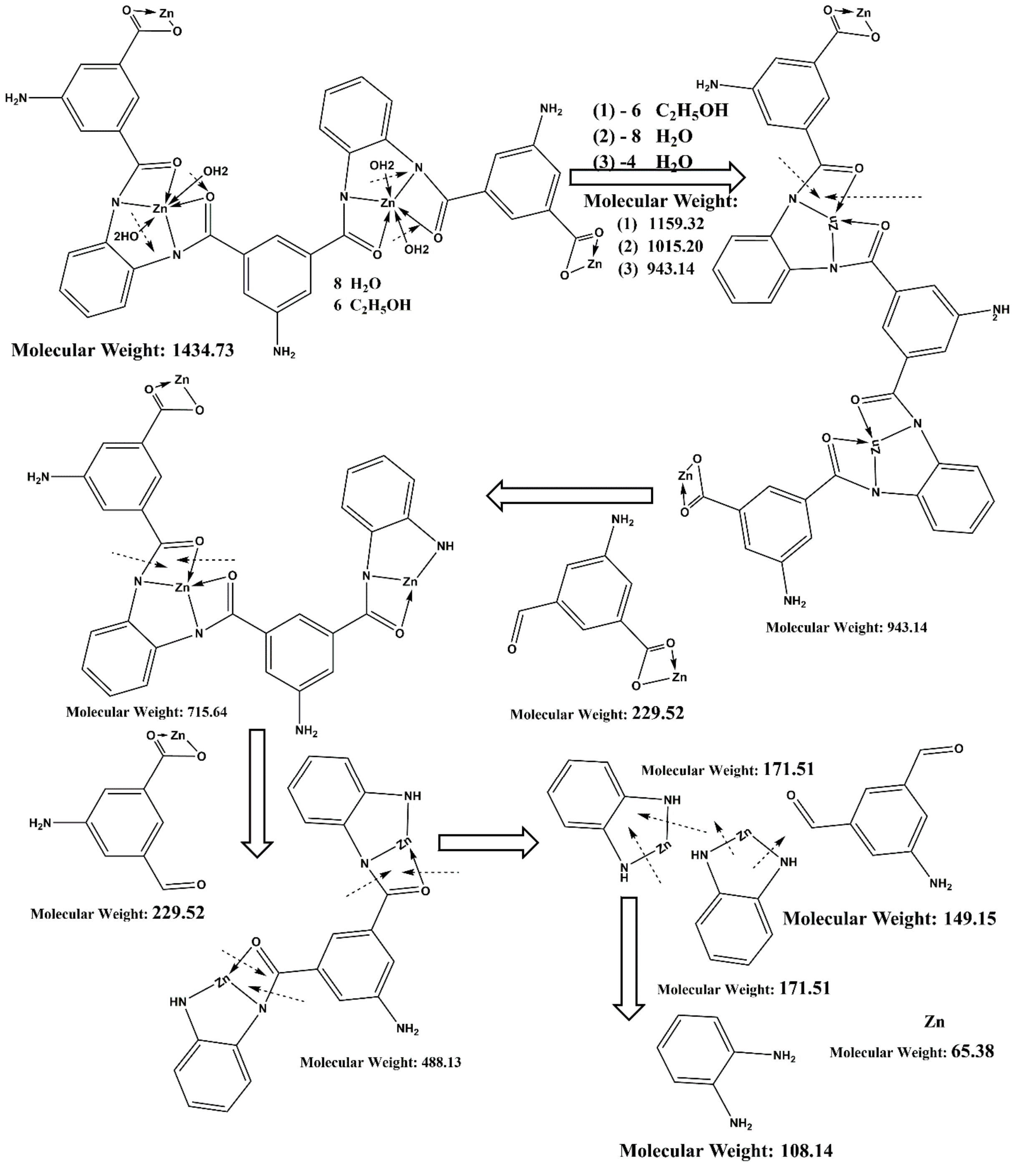

Fig. S3: The proposed fragmentation Scheme of the Zn-MOFs-NP .

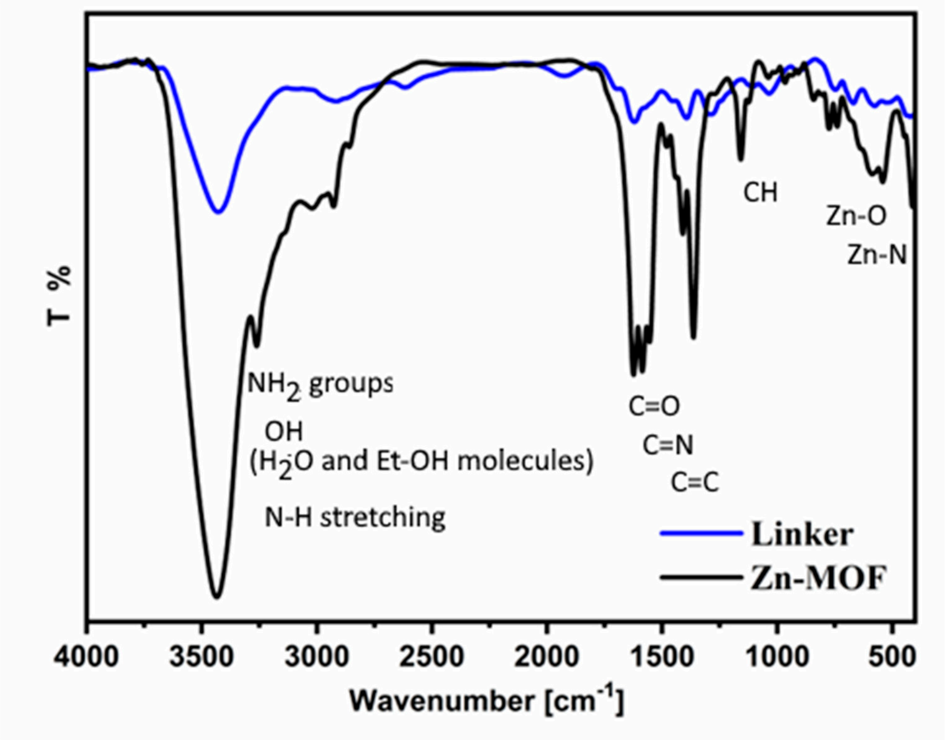

Fig. S4: FT-IR spectra of Zn-MOFs-NP and nano organic linker.

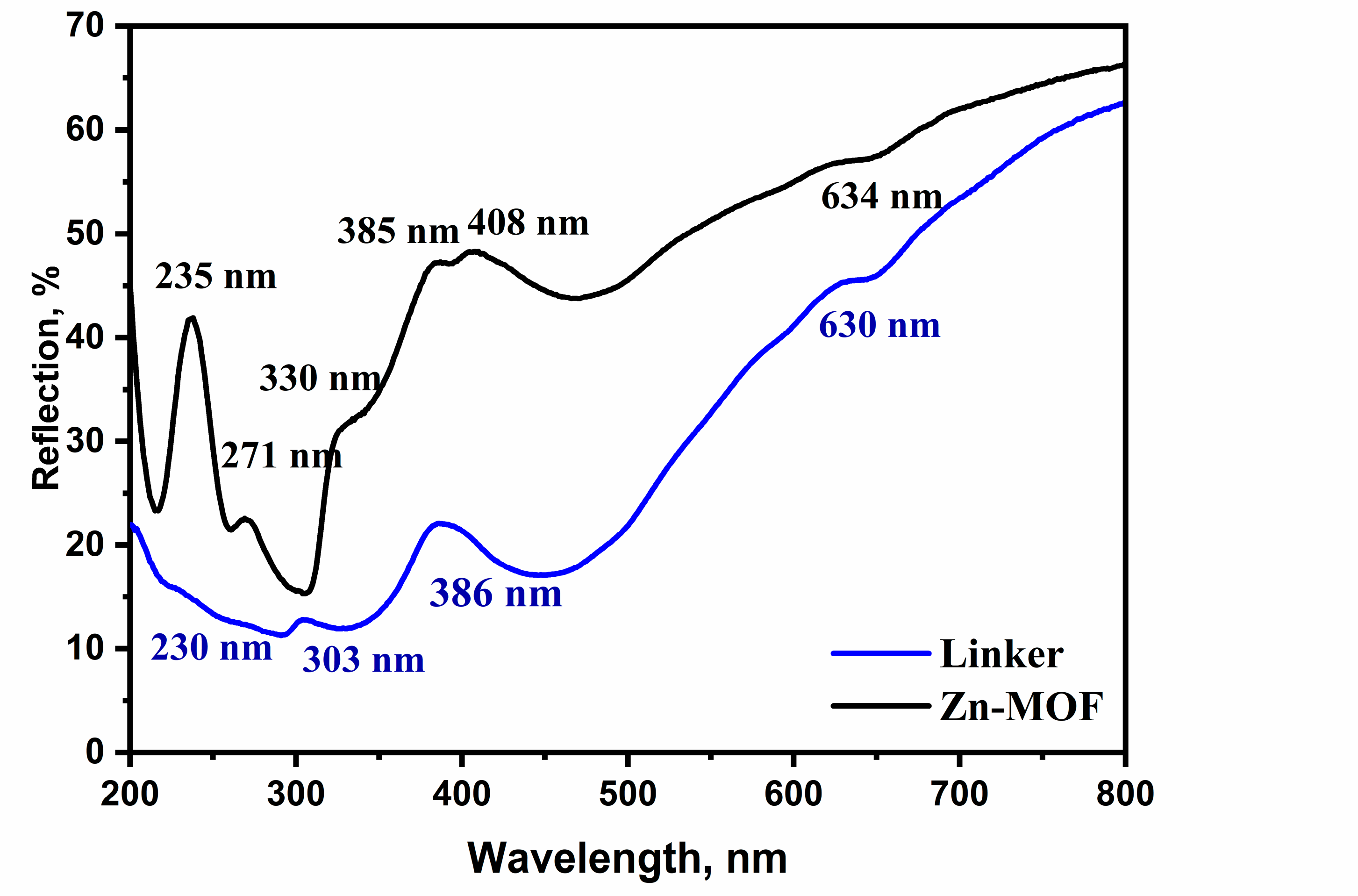

Fig. S5: The electronic-reflection spectra of Zn-MOFs-NP and nano organic linker.

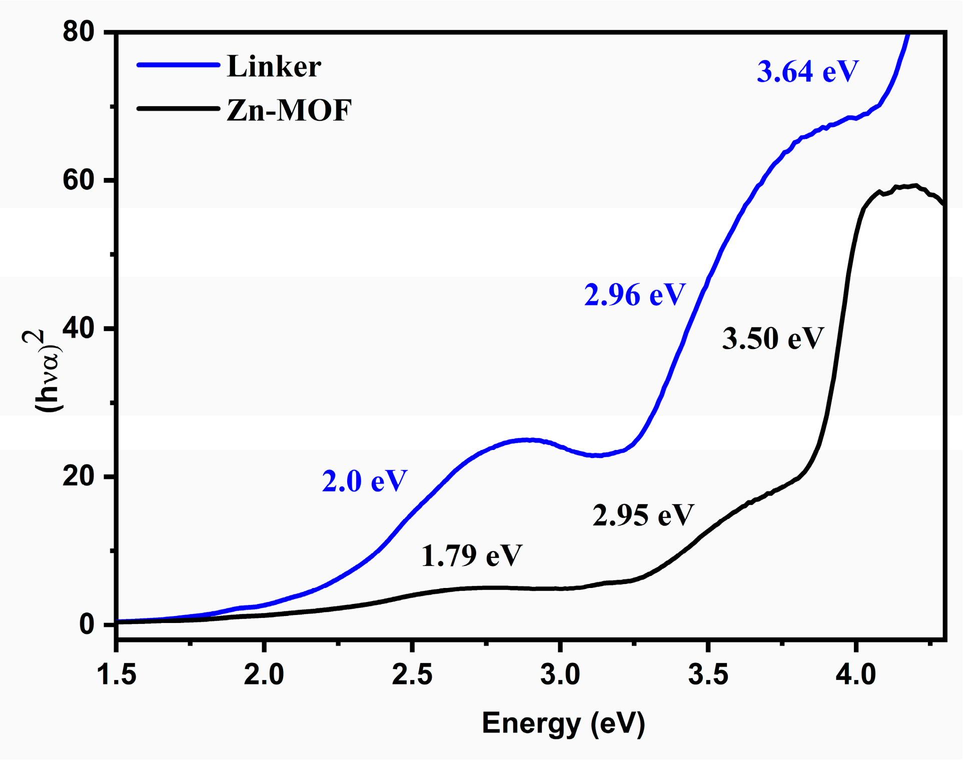

Fig. S6: The bandgap energy spectra of Zn-MOFs-NP and nano organic linker.

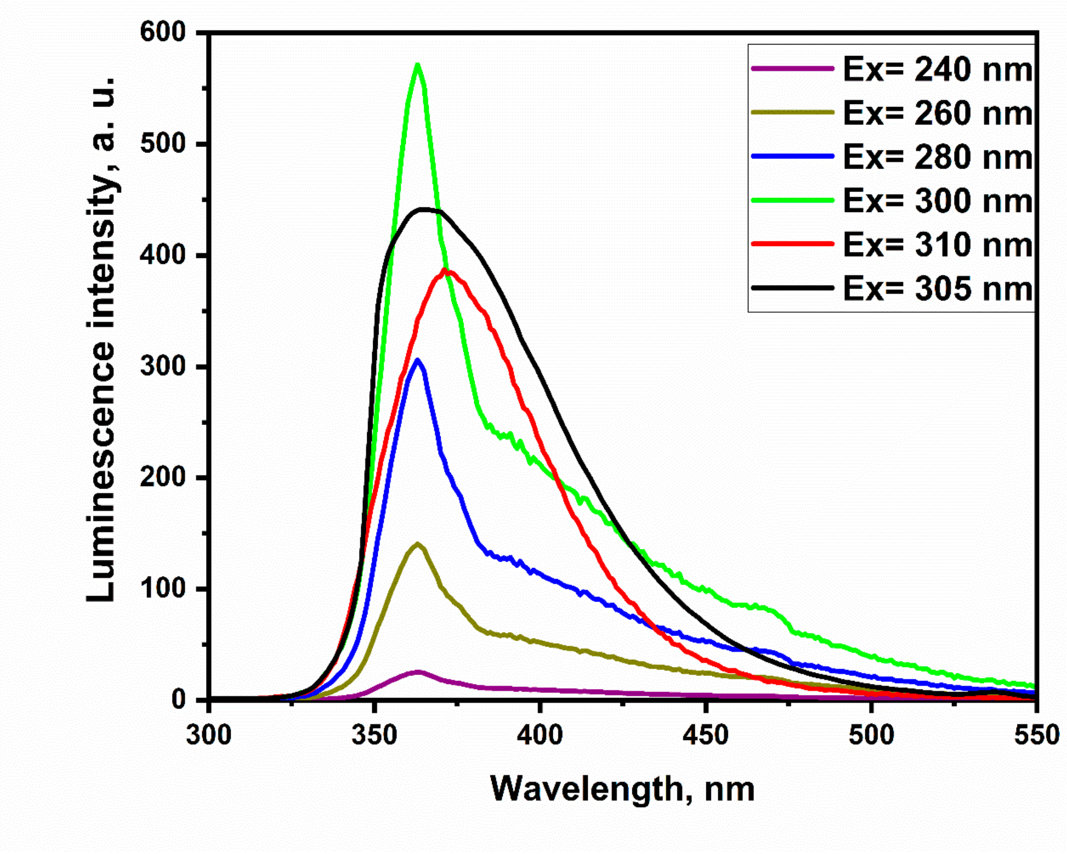

Fig. S7. The PL emission spectra at different excitation wavelength for the Zn-MOFs-NP.

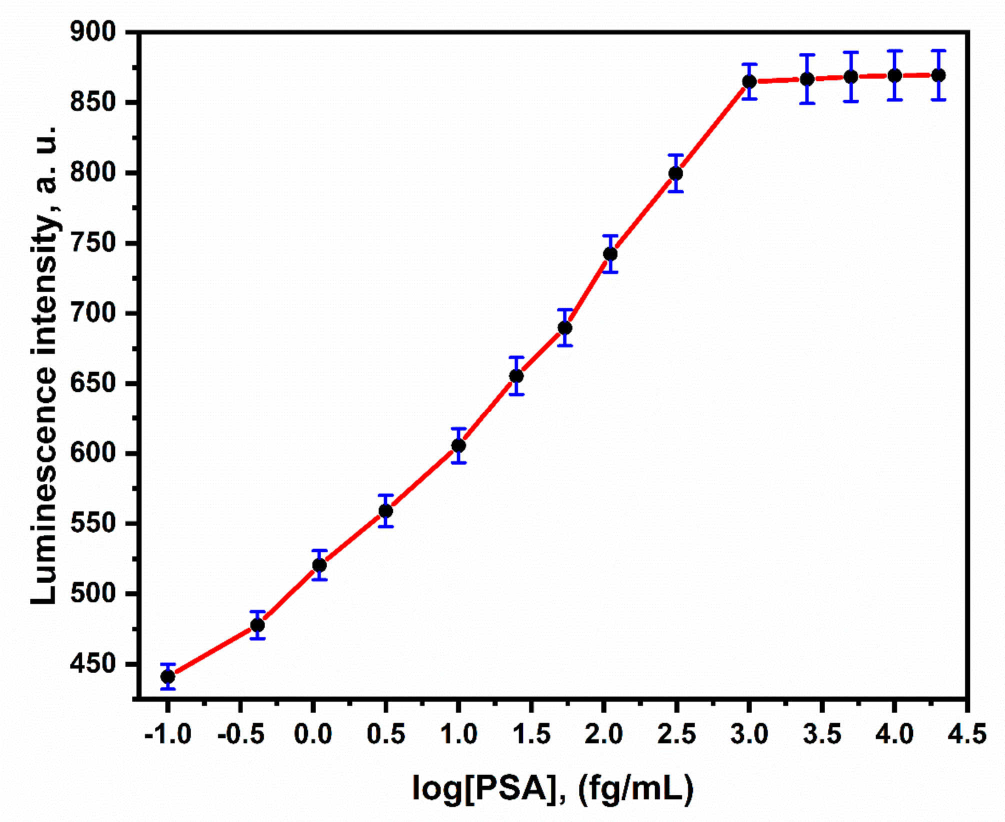

Fig. S8. A full relationship (calibration graph) between the PL intensity of Zn-MOFs-NP and the logarithm PSA concentration (log [PSA]).

Table S1: Summary of XRD data, Miller indices and interplanar distances of Zn-MOFs-NP.

**Peak**	**2θ**	**Intensity**	**Intensity**	**d-value**	**θ(Radians)**	**Sin θ**	**Sin² θ**	**Ratio 1**	**Ratio 2**
No.		Count	%	A°					
1	11.411	13.1	4.055728	7.748306848	0.09958	0.09942	0.00988	1	2
2	13.25	162	50.1548	6.676745483	0.115628	0.11537	0.01331	1.3467	2.693481
3	14.65	64	19.81424	6.041692205	0.127845	0.1275	0.01626	1.2213	2.442544
4	15.603	54.4	16.84211	5.674756376	0.136162	0.13574	0.01843	1.1335	2.267006
5	17.448	20.8	6.439628	5.07862653	0.152263	0.15167	0.02301	1.2485	2.497077
6	19.988	266	82.35294	4.438617001	0.174428	0.17355	0.03012	1.3092	2.618347
7	21.504	35.6	11.02167	4.129003516	0.187658	0.18656	0.0348	1.1556	2.311186
8	22.788	323	100	3.899173527	0.198863	0.19755	0.03903	1.1214	2.242722
9	23.679	13.7	4.241486	3.754432173	0.206638	0.20517	0.0421	1.0786	2.157181
10	24.406	38.2	11.82663	3.644217397	0.212983	0.21138	0.04468	1.0614	2.122804
11	25.638	59.8	18.51393	3.471823825	0.223734	0.22187	0.04923	1.1018	2.203551
12	27.698	267	82.66254	3.218112736	0.241711	0.23936	0.0573	1.1639	2.327785
13	29.326	59.2	18.32817	3.043061223	0.255918	0.25313	0.06408	1.1184	2.236717
14	29.736	39.4	12.19814	3.002030139	0.259496	0.25659	0.06584	1.0275	2.055045
15	32.177	45	13.93189	2.779644115	0.280797	0.27712	0.0768	1.1664	2.332823
16	33.166	27	8.359133	2.698980953	0.289428	0.2854	0.08146	1.0607	2.121333
17	33.979	16.1	4.98452	2.636240125	0.296523	0.2922	0.08538	1.0482	2.09633
18	34.669	67.3	20.83591	2.585335553	0.302544	0.29795	0.08877	1.0398	2.079534
19	35.448	28.6	8.854489	2.53028521	0.309342	0.30443	0.09268	1.044	2.087973
15	36.218	41.7	12.91022	2.478237545	0.316062	0.31083	0.09661	1.0424	2.08489
16	36.828	29.3	9.071207	2.438577466	0.321385	0.31588	0.09978	1.0328	2.065583
17	38.409	44.2	13.68421	2.34175864	0.335182	0.32894	0.1082	1.0844	2.168797
18	40.134	39.5	12.2291	2.244995302	0.350235	0.34312	0.11773	1.0881	2.176123
19	40.405	37.4	11.57895	2.230560897	0.3526	0.34534	0.11926	1.013	2.025969
20	41.046	42.1	13.03406	2.197195	0.358194	0.35058	0.12291	1.0306	2.061204
21	42.409	50.4	15.60372	2.12967846	0.370088	0.3617	0.13083	1.0644	2.128821
22	43.85	13.1	4.055728	2.062976118	0.382663	0.37339	0.13942	1.0657	2.131423
23	44.542	34.7	10.74303	2.032519144	0.388702	0.37899	0.14363	1.0302	2.060388
24	45.047	32.4	10.03096	2.010899889	0.393109	0.38306	0.14674	1.0216	2.043235
25	45.424	30.2	9.349845	1.995082872	0.396399	0.3861	0.14907	1.0159	2.031838
26	46.762	19.7	6.099071	1.941067303	0.408075	0.39684	0.15748	1.0564	2.11286
27	47.726	21.6	6.687307	1.904085774	0.416488	0.40455	0.16366	1.0392	2.078443
28	49.6	37.7	11.67183	1.83644338	0.432842	0.41945	0.17594	1.075	2.150047
29	52.148	32.8	10.1548	1.752547434	0.455077	0.43953	0.19319	1.098	2.196067
30	53.014	33.4	10.34056	1.725941441	0.462634	0.44631	0.19919	1.0311	2.062137
31	56.958	19.1	5.913313	1.615437992	0.497052	0.47684	0.22737	1.1415	2.282977
32	58.465	20.3	6.28483	1.577337031	0.510203	0.48835	0.23849	1.0489	2.097788
33	63.867	14	4.334365	1.456323641	0.557345	0.52893	0.27977	1.1731	2.34619

Table S2: Summary of calculated crystallite size of Zn-MOFs-NP at different position on XRD patterns

Position	Area	Cry Size L(nm)	Microstrain	RMS Strain(%)
11.38552	4.855149	135.7	0.1	0.1
13.23369	43.44118	123.9	0.1	0.1
14.63309	16.7502	114.3	0.1	0.1
15.58916	18.16841	151.4	0.1	0.1
17.43039	7.355614	139.9	0.1	0.1
19.95442	83.19238	156	0.1	0.1
21.48416	9.773613	158.9	0.1	0.1
22.76481	121.5275	146.3	0.1	0.1
24.37391	12.47938	145.6	0.1	0.1
25.55095	31.52574	123.9	0.1	0.1
27.65815	91.70529	151.6	0.1	0.1
29.44304	44.44398	118.1	0.1	0.1
32.1089	16.86871	142.2	0.1	0.1
33.10664	10.92236	130.1	0.1	0.1
34.65973	42.66779	122.5	0.1	0.1
36.2363	37.32982	110.7	0.1	0.1
38.35792	16.15865	148	0.1	0.1
40.30312	27.3514	123.4	0.1	0.1
41.01343	16.14014	140.8	0.1	0.1
42.34565	16.44194	159.2	0.1	0.1
44.85477	46.3486	108.4	0.1	0.1
46.70172	9.631201	129.7	0.1	0.1
47.67431	7.208591	101.2	0.1	0.1
48.37956	10.19796	123.3	0.1	0.1
49.60343	21.3051	127.4	0.1	0.1
52.09599	20.96037	121	0.1	0.1
52.9366	24.26174	119.3	0.1	0.1
57.0886	77.75088	113	0.1	0.1
65.37826	43.0985	113	0.1	0.1

Table S3: Sensitivity and regression parameters for Zn-MOFs-NP chemosensor

**Parameter**	**Method**
λ_{ex}, nm	305
λ_{em}, nm	372
Linear range, fg/mL	0.1 – 20000
Limit of detection (LOD), fg/mL	0.145
Limit of quantification (LOQ), fg/mL	0.438
Regression equation	(Y=a+bX)*
Intercept (a)	523.01
Slope (b)	105.21
Standard deviation	4.611
Correlation coefficient (r^2)	0.983

**Y, is photoluminescence intensities; X, is the concentration of- PSA in fg/mL; a, is intercept; b, is slope.*
